# Sensory substitution: The affordance of passability, body-scaled perception, and exploratory movements

**DOI:** 10.1371/journal.pone.0213342

**Published:** 2019-03-27

**Authors:** Carlos de Paz, David Travieso, Jorge Ibáñez-Gijón, Miguel Bravo, Lorena Lobo, David M. Jacobs

**Affiliations:** 1 Facultad de Psicología, Universidad Autónoma de Madrid, Madrid, Spain; 2 Facultad de Ciencias de la Salud y de la Educación, Universidad a Distancia de Madrid, Madrid, Spain; University of Minnesota, UNITED STATES

## Abstract

The theory of affordances states that perception is of environmental properties that are relevant to action-capabilities of perceivers. The present study illustrates how concepts and methodological tools from the theory of affordances may help to advance research in the field of sensory substitution. The sensory substitution device (SSD) that was used consisted of two horizontal rows of 12 coin motors that each vibrated as a function of the distance to the nearest object. Sixty blindfolded participants used the SSD to explore virtual horizontal apertures with different widths. They were asked to judge the passability of the apertures. Participants with narrow shoulders judged narrower apertures as passable than participants with wide shoulders. This difference disappeared when aperture width was scaled to shoulder width, demonstrating that perception was body scaled. The actual aperture width was closely related to aspects of the exploratory movements and to aspects of the vibrotactile stimulation that was obtained with the exploratory movements. This implies that the exploratory movements themselves and the vibrotactile stimulation were both informative about the aperture width, and hence that the perception of passability may have been based on either of them or on a global variable that spans vibrotactile as well as kinaesthetic stimulation. Similar performance was observed for participants who accomplished the 7-trial familiarization phase with or without vision, meaning that practice with vision is not indispensable to learn to use the SSD.

## Introduction

Sensory substitution devices (SSDs) are devices that substitute one perceptual modality (usually vision) with another one (usually hearing or touch) [1–7]. A substantial number of SSDs and experiments with SSDs have been oriented toward the recognition of objects and the perception of properties of objects such as their shape or size [8–13]. Our research is more aligned with SSD-based studies that address action-related tasks such as locomotion toward targets [14, 15], collision avoidance [16, 17], stepping on ground-level objects [18], or the perception of the direction and location of objects [19–21]. Relatedly, we believe that it would be fruitful to base research in the field of sensory substitution on the inherently action-related theory of affordances. Affordances are defined as properties of the environment that provide possibilities for action to a given organism [22–25]. The concept of affordance is a cornerstone of the ecological approach to perception and action, which was initiated by Gibson [22]. The ecological approach claims that, rather than abstract, organism independent properties of the environment, individuals perceive affordances, which is to say, they perceive what actions the environment affords them.

Previous research in the field of sensory substitution that has applied the theory of affordances includes studies about the perception of the climbability of stairs [26] and the passability of apertures [27–30]. The present study concerns the passability of apertures. To describe the purpose of our study, we first need to review classic affordance-based research on the visual perception of passability (e.g., [31–35]). In their pioneering study, Warren and Whang [35] asked two groups of individuals, with narrow and wide shoulders, to judge the passability of apertures (their Experiment 2). The aperture width that was judged as passable on 50% of the trials was referred to as the critical aperture width, and denoted as A_c_. This critical width was smaller for the narrow-shoulders groups than for the wide-shoulders group. In a next step of their analysis, Warren and Whang [35] calculated dimensionless numbers, known as π-numbers, as the ratio of aperture width over shoulder width. The critical value of this number, π_c_, was again defined as the value that leads to 50% of affirmative judgments. Warren and Whang [35] showed that the differences between individuals with narrow and wide shoulders disappeared in the body-scaled analysis: for both groups, the value of π_c_ was 1.16. This observation has been highly influential in the literature on the visual control of action, at least in part because body-scaled descriptions of the environment are more useful to organisms in the control of action than descriptions in extrinsic units.

Body-scaled descriptions of the environment should be expected to be equally important in sensory substitution as in the visual control of action. Even so, body-scaled values have only been reported in two SSD-based studies [26, 29]. Of these two, only the recently published study by Favela et al. [29] addressed the passability of apertures. The authors of [29] calculated the π_c_ values for the passability perceived visually, with a cane, and with an SSD (the Enactive Torch; based on a single vibrotactile motor). An overall π_c_ value of 1.36 was observed. The differences in the π_c_ values for the different perceptual modalities did not reach significance, hence suggesting that perceiving the affordance with the cane and with the SSD is in some sense equivalent to perceiving the affordance through regular vision. The authors of [29] did not analyze the movements that the SSD users performed during the perception of the apertures. The exploratory movements that underlie the perception of apertures with SSDs have not been addressed in other studies either.

The importance of exploration in sensory substitution, however, has often been demonstrated with other tasks. Examples of this can be found in studies that address the localization of targets [20, 21] and orienting and walking to targets [15, 19]. Exploration with SSDs is crucial, among other reasons, because it leads to rich sensory flows and because it permits users to exploit sensory-motor contingencies [36]. The most relevant study with regard to exploration in the SSD-based perception of apertures is the study reported by Kolarik et al. [30]. These authors compared the action of walking through an aperture using vision and using one of two auditory SSDs (the K-sonar and the Miniguide; based on echolocation). When participants used the SSDs, they moved slower on average and with lower peak velocities, and they rotated their shoulders more (cf. [37, 38]). Although the study by Kolarik et al. [30] analyzed movement variables, these variables were related to the action of passing through apertures more than to the perceptual exploration of the apertures. Given the demonstrated importance of exploration in other sensory substitution tasks, it seems relevant to analyze the exploratory movements that underlie the perception of passability with SSDs.

Another fundamental issue for research in the field of sensory substitution is the process of learning to use SSDs [18, 20, 39, 40]. A substantial number of studies on sensory substitution included practice phases in which participants used SSDs together with regular vision. Given that SSDs are often intended to be used by visually impaired individuals, it is relevant to ask if participants can familiarize themselves with the devices during practice without vision. Several studies have shown that visually impaired individuals can indeed make use of SSDs [39–42]. Furthermore, in [18] we presented an SSD on the lower leg and showed that practice without vision led to larger improvements in performance than practice with vision. In the specific case of the perception of passability, the reviewed studies include practice phases with [30] and without vision [27–29]. It is impossible to compare those practice conditions, however, due to the different experimental designs and SSDs used in the studies.

In the present study, then, we analyzed three aspects of the SSD-based perception of the passability of apertures. First, we analyzed the critical A_c_ and π_c_ values for blindfolded SSD users. We hypothesized that the critical aperture values, A_c_, would be different for individuals with narrow and wide shoulders, but that the corresponding body-scaled values, π_c_, would not, as in studies on visual perception [35]. Furthermore, based on the results of [29], we hypothesized that the π_c_ values for SSD users would not differ significantly from the ones previously observed for visual tasks (cf. [26]). Second, we analyzed the exploratory movements that underlie SSD-based perception. In this sense, it is relevant to note that we used a psychophysical estimation task, without asking individuals to actually walk through the apertures. This means that the observed movements can be attributed to perceptual exploration. We expected more pronounced exploratory movements for wider apertures, because, with our SSD, such more pronounced movements would be necessary to detect both edges of the wider apertures. Third, we compared two brief practice conditions in which users explored the apertures with the SSD and with regular vision or with the SSD and a short wooden rod. On the basis of [18], we hypothesized that practice with vision is not indispensable and, hence, that the task can also be performed after practice without vision.

## Materials and methods

### Participants

Sixty participants (53 women and 7 men) performed the experiment. Their ages ranged from 18 to 23 years (*M* = 19.8; *SD =* 1.2). All of them had normal or corrected to normal vision and normal motor capacities. None of the participants had previous experience with SSDs. Their shoulder width, measured with an anthropometric ruler, ranged from 37 to 47 cm (*M* = 41.5; *SD =* 2.42). Participants were divided into two groups, with narrow and wide shoulders, using the median shoulder width of 41 cm as cut-off point. Participants signed an informed consent form prior to the experiment. They received course credit for their participation. The research protocol was approved by the research ethics committee of the Universidad Autónoma de Madrid (CEI 52–957).

### Apparatus

**Fig 1:**
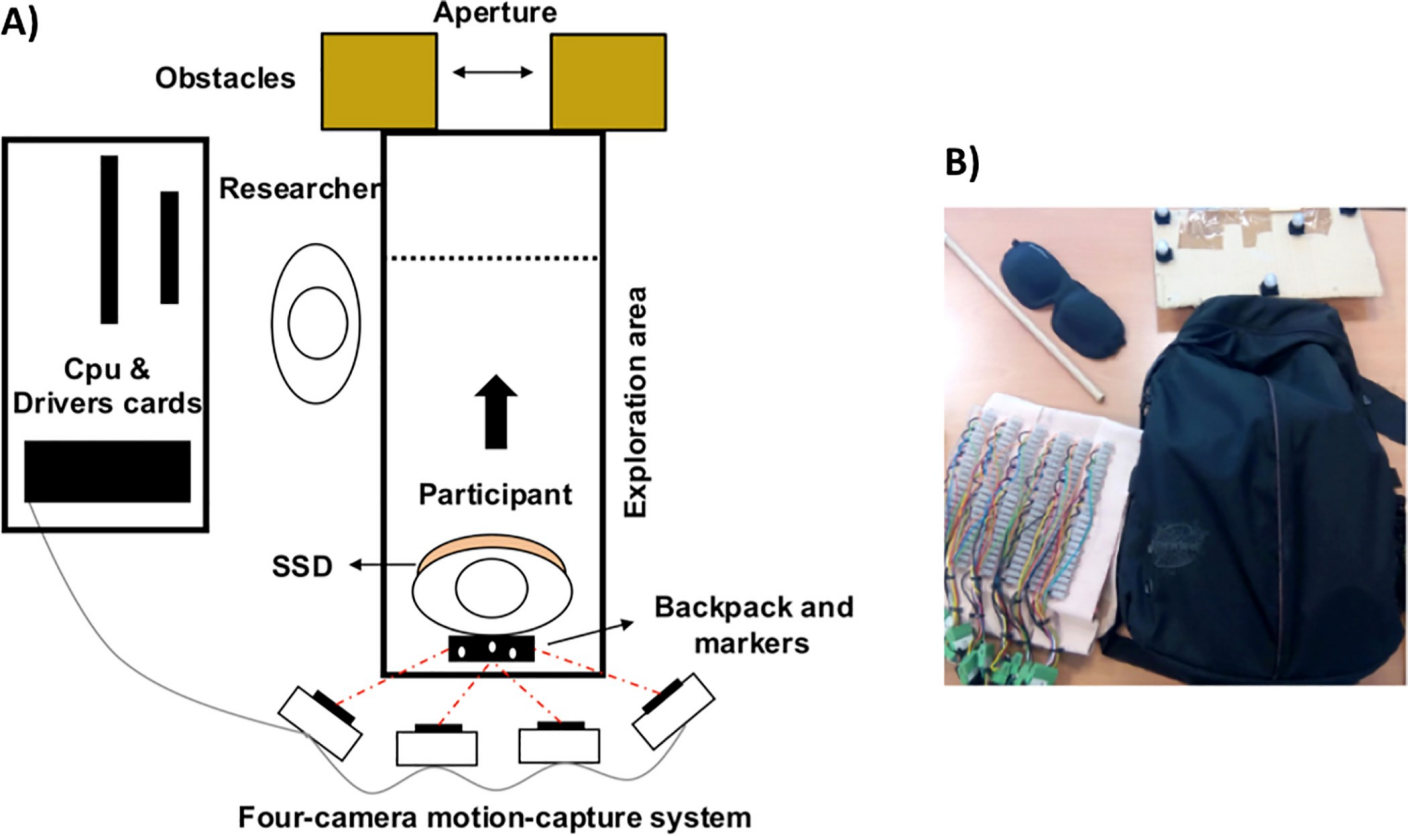
Experimental material. A. Schematic drawing of experimental setup. B. From upper left to lower right corner: wooden rod, blindfold, reflective markers, elastic band with motors, and backpack with microcontrollers and battery.

The experimental setup is shown in Fig 1A. The setup included an exploration area of 200 × 80 cm (depth × width). At the end of this area, a physical aperture was created with two cardboard obstacles of 152 × 35 × 43 cm (height × depth × width). This physical aperture was needed only in the practice trials that preceded the actual experiment, in which it was explored either through vision or with a wooden rod. A virtual aperture that closely matched the physical one determined the vibration of the SSD. The virtual aperture differed from the physical one in the sense that it was two-dimensional and in the sense that it extended infinitely to the left and right (as did the visual aperture in the *wall* condition in [43]). More precisely, the virtual aperture was formed by two virtual wall segments at the end of the exploration area in a way that the virtual wall segments coincided with the surface of the cardboard boxes at the side of the participant. The virtual aperture could not be seen or touched; it was used only to compute the pattern of vibration. The center of the aperture remained aligned with the center of the exploration area throughout the experiment. A four-camera motion-capture system (Qualisys Inc., Sweden) registered the position and movement of the participant.

Different parts of the SSD are illustrated in Fig 1B. The SSD included an elastic band with 6 rows of 12 vibrotactile coin motors (with a diameter of 12 mm; not shown in the figure). Only the middle 2 rows of 12 motors were activated in this experiment (see [44], for an experiment in which all 6 rows were used). The horizontal distance between consecutive motors was 1.5 cm. The vertical distance between the used rows was 2.5 cm. The elastic band with the motors was attached to the abdomen with hook and loop fasteners. To compute the activation levels of the motors, we ignored the fact that the elastic band curved around the abdomen: the two rows of motors were assumed to lie in a plane. Using this assumption, each motor vibrated as a function of the distance to the first-encounter object along a line that pointed from the considered motor position outward, in a horizontal direction parallel to the sagittal plane. Hence, a vibration was produced whenever the position and orientation of the participant were such that the line of sensitivity associated to a motor pointed toward one of the wall segments that formed the virtual aperture. This vibration was more intense if the participant was nearer to the wall segment. In contrast, no vibration occurred when a motor pointed toward the opening between the virtual wall segments. An animated video of a single trial that shows the functioning of the device and the typical behavior of participants is provided in the supporting information (S1 Video).

To achieve that the motors vibrated as a function of their spatial relation to the virtual aperture, the information from the motion-registration system was sent to a computer (PC Intel Core i7, 3.07 GHz) and processed on-line with MATLAB software (The MathWorks, Inc., Natick, 2016). MATLAB calculated if the motors pointed toward the virtual walls or toward the opening between them. If a motor pointed toward the virtual walls, the distance along the line of sensitivity between the motor and the wall was determined. This distance was converted into a vibration level with the following equation: *VL* = 100—*c* × *D*_obstacle_, where *VL* is the vibration level of a motor expressed as a percentage of the maximal vibration level, *D*_obstacle_ is the distance between a motor and the wall (in cm), and *c* is a parameter that determines the scaling between distance and vibration level. We used *c* = .25, meaning that the vibration level decreased with approximately 25% for increases in distance of 100 cm. Based on our experience with the task, whether a motor vibrated or not was more important than the precise level of vibration. The relation between the vibration level and empirically measured frequencies of vibration can be found in [44].

Pro-mini Arduino microcontrollers were used to control the motors. The information about the vibration levels was sent from the computer to the microcontrollers with a wireless Xbee device (model S2). A NiMh Battery of 4000 mA/h supplied the energy for the motors. The microcontrollers and the battery were carried in the backpack.

### Design

Two between-subjects variables were used. The first one, shoulder width, was based on the division in groups described in the Participants subsection. The second between-subjects variable was the type of practice that participants received. During the practice trials, in addition to the stimulation provided by the SSD, half of the participants explored the aperture with a 20-cm long wooden rod whereas the other half used regular vision. As a result, we had 4 groups of 15 participants: 2 (shoulder width) × 2 (practice modality) x 15 (participants per group) = 60 participants. A single within-subjects variable was used: aperture width. The apertures ranged from 30 to 90 cm in steps of 10 cm. During the practice phase, each of the seven apertures was used once, leading to a 7-trial practice. During the experimental phase, each aperture was repeated three times, resulting in 21 experimental trials. The order of the trials was randomized per participant.

### Procedure

At the beginning of the experiment, participants received the following written instructions: “Your task in this experiment will be to estimate if the aperture between two obstacles is large enough to allow you to walk through it without shoulder turns. You will receive stimulation provided by our SSD. In each trial, you will start from the same initial position and you will have 30 s to freely explore the aperture. When this time is over, you will be asked to respond yes or no.” After the instructions, participants performed the seven practice trials. Those who performed the practice trials with the wooden rod were blindfolded, whereas those who performed the practice trials with regular vision were not. During the experimental trials, all participants were blindfolded. During those trials, participants received a verbal instruction to stop whenever they came closer than 50 cm to the aperture. To help the experimenter with these instructions, the line corresponding with a distance of 50 cm was clearly marked on the floor. Participants were asked to make a forced-choice judgment concerning the passability of the apertures after 30 s. No feedback was given. Before the following trial, one of the experimenters helped the participants back to the initial position. The experiment was performed in a single session that lasted about 45 min.

### Data acquisition and preprocessing

The experimenters manually registered the judgments of the participants about the passability of the apertures. Movement data were collected with the Qualysis motion-registration system at 120 Hz, with a position error of less than 1 mm. Five reflective markers attached to the participant’s back were used to define a rigid body in Qualysis. The position and orientation signals of the rigid body that were imported from Qualysis were filtered in MATLAB with a 4th order low-pass Butterworth filter with a cut-off frequency of 8 Hz.

The observed movements typically consisted of two qualitatively distinct phases. First, a phase of translation during which the participant moved from the starting point toward the aperture. Second, a phase of exploration in which participants rotated the upper body back and forth in front of the virtual aperture, with much less body translation than in the first phase. Only the exploration phase was considered in the analyses. To this end, the time series of each trial was divided in translation and exploration phases using the first prominent negative peak of the forward acceleration after the participant moved 50 cm in the direction of the aperture.

### Analysis and dependent variables

#### Critical numbers

To determine the critical aperture width, A_c_, we fitted logistic functions to the proportion of affirmative answers of each participant using the equation:

$$P(passable)=\frac{1}{1+e^{-(a+bx)}},$$

in which *x* is the aperture width and *a* and *b* are parameters that are related to the slope and intercept, respectively. At this model-fitting stage, we used Pearson correlations between the observed proportions of affirmative judgments per aperture width and the proportions predicted by the fitted logistic function (i.e., the height of the function) as an indication of the goodness of fit of the function (see Equation 5.8 of [45]). Participants with *r*^2^s lower than .60 (*n* = 11) were removed from all subsequent analyses. Varying the *r*^2^ cut-off between .40 and .90 did not substantially change any of the statistical results reported in this article.

The critical value A_c_ was established as the aperture width at which the fitted logistic function reached the proportion of 0.5. An analytic expression of the critical value (A_c_ = -*a* / *b*) can be derived from the equation by setting *P*(*passable*) = .5 and solving for *x*. To obtain body-scaled measures, we computed the ratio of aperture width over shoulder width for each participant. The same logistic fits were performed on the body-scaled data as on the original data, hence obtaining the critical values π_c_ as the points where the body-scaled logistic functions reached the proportion of 0.5. The main dependent variables in this set of analyses, then, were the critical values A_c_ and π_c_. In addition, we included the parameters *a* and *b* of the original logistic fits in the analyses.

#### Exploratory movements

**Fig 2:**
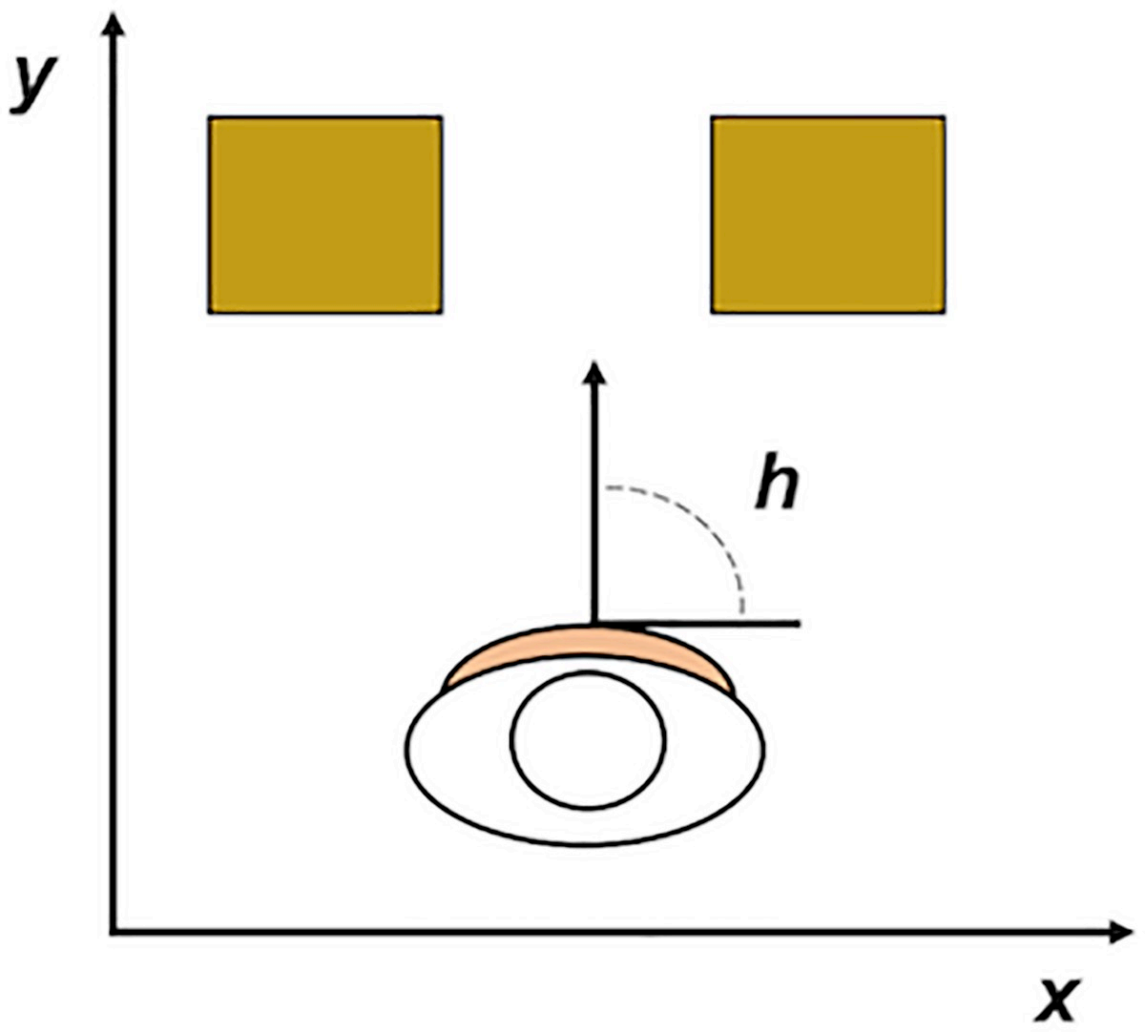
Variables used to compute exploratory movement variables. *x* = horizontal position; *y* = depth position; and *h* = heading direction.

The dependent variables related to the exploratory movements were computed from the time series of the horizontal and depth position of participants and their heading direction (*x*, *y*, and *h* in Fig 2). Using these variables, we first computed the total linear displacement during the exploration phase of a trial. Intuitively, trials with more displacement led to higher values of this variable. Said with more precision, the total linear displacement was computed as the integral over time of the translation, without taking into account the height dimension, and independent of the direction of the translation in the *x*-*y* plane. To characterize the rotational component of the exploration we defined the total angular displacement as the integral over time of the changes in heading direction. The total linear and angular displacements were chosen because they provide simple descriptions of the spatial extent of the exploratory movements. Related measures of this spatial extent (such as the range of translational or angular movement or the movement variability) led to similar results as the variables reported in the article.

Another relevant aspect of the exploratory movements in this task is how close to the aperture the exploratory oscillations were performed. We operationalized this aspect of the exploration with the minimal distance to the aperture throughout a trial (i.e., the lowest value of *y* during the 30 s of exploration). Finally, as a simple measure that captures the exploration-induced patterns in vibratory stimulation, we computed the average number of active (columns of) motors during a trial. The theoretical minimum and maximum of this variable were 0 (all motors silent during the entire trial; possible only if a participant would continuously point toward the gap between the obstacles) and 12 (all motors active during the entire trial; possible only if a participant would continuously point toward an obstacle). To summarize, the dependent variables that we used in this set of analyses were the linear displacement, angular displacement, minimal distance to the aperture, and the average number of active motors.

#### Type of errors

The variable type of error was defined as a categorical variable with three levels: false positive judgments (positive answers for apertures narrower than the critical width), correct judgments, and false negative judgments (negative answers for apertures wider than the critical width). We tested the effect of this independent variable on all four exploration-related dependent variables that were described in the previous subsection. Obviously, wide apertures cannot lead to false positive judgments whereas small apertures cannot lead to false negative judgments. This uninteresting relation between type of error and aperture width may give rise to a bias, in part because our dependent variables are also related to aperture width. To avoid this bias, the dependent variables were normalized. Variables that increased with aperture width were divided by the aperture width per trial so as to obtain a ratio per cm of aperture (e.g., for the total displacement, the ratio would indicate how many centimeters were traversed during the exploration phase of the trial per centimeter of aperture). Variables that decreased with aperture width were multiplied with the aperture width. We hence obtained four aperture-normalized dependent variables in the set of analyses concerning the type of error.

### Statistical analysis

Differences in previously described dependent variables were assessed with Analyses of Variance (ANOVAs). All dependent variables were averaged over the three repetitions of trials per individual before the ANOVAs were applied. Greenhouse-Geisser corrections were reported when needed. The full dataset can be found in the supporting information (S1 Dataset).

## Results

### Critical numbers

**Fig 3:**
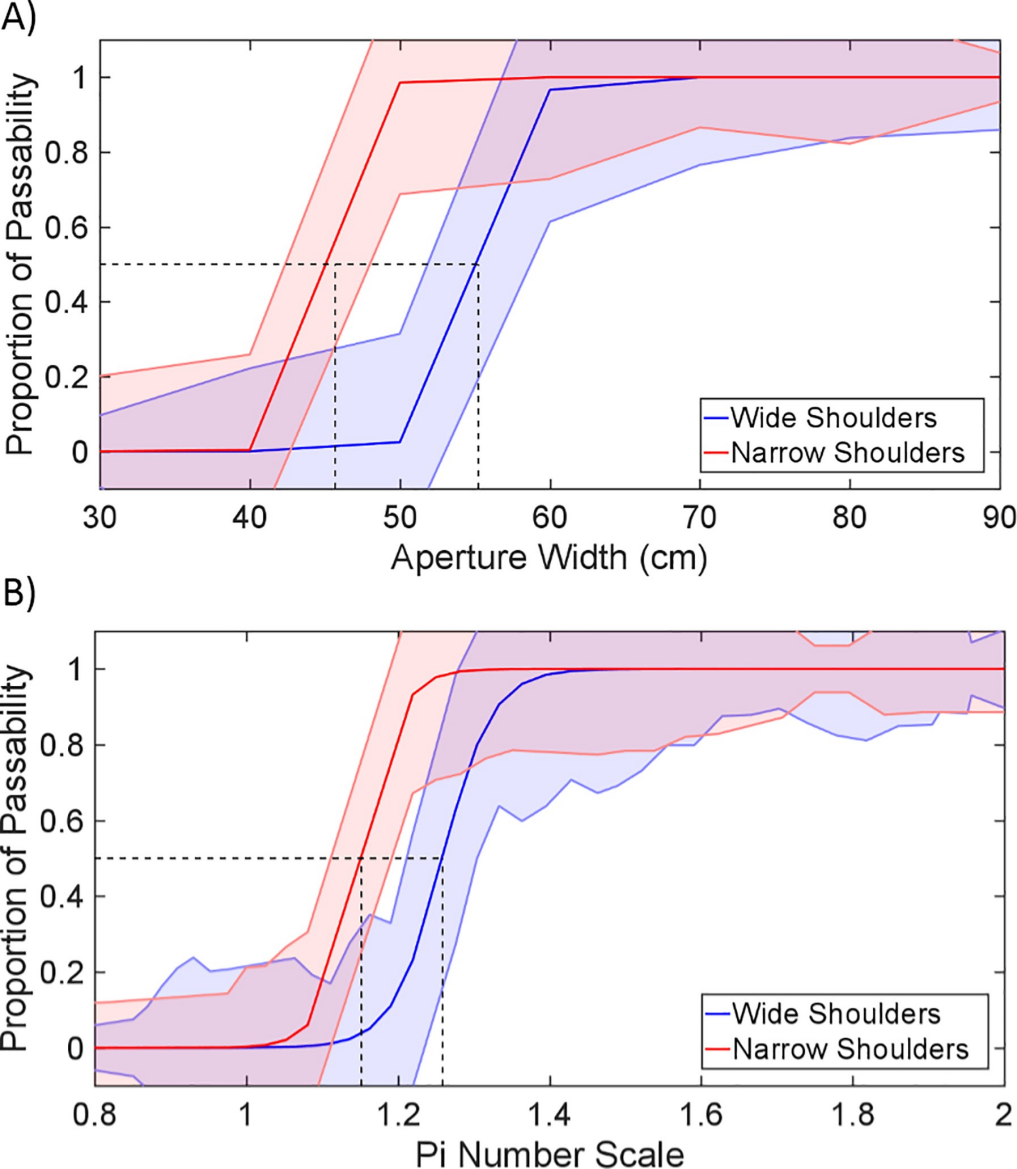
Logistic fits on proportions of affirmative judgments. A. Fitted proportions for both shoulder-width groups as a function of aperture width. B. Fitted proportions for both groups as a function of π-number (aperture width / shoulder width). The edges of the colored regions indicate the logistic fits ± one standard deviation.

Table 1 shows the results of the ANOVAs on the critical numbers and the parameters of the logistic fits. The independent variables in these analyses were shoulder width and type of training. The only significant effect was the effect of shoulder width on the critical aperture width A_c_. The values of A_c_ were lower for participants with narrow shoulders (*M =* 47.6 cm; *SD* = 9.5) than for participants with wide shoulders (*M =* 53.9 cm; *SD =* 8.8). This effect was not significant when the apertures were body scaled (i.e., for π_c_). Fig 3 presents logistic fits with averaged parameters for the original and body-scaled analyses. We performed a *t* test to check if our overall π_c_ value (*M* = 1.22; *SD* = .23) differed from the value of π_c_ = 1.16 in Experiment 2 of Warren and Whang [35]. The difference was not significant, *t(*48) = 1.82, *p* = .08. As can be seen in Table 1, the effect of training and the interaction were not significant for any of the dependent variables. Likewise, no significant effects on the slope and intercept parameters of the logistic fits were observed.

**Table 1:** Results of ANOVAs on critical numbers and parameters of logistic fits.

	Shoulder Width			Training			Shoulder Width x Training		
	*F*_1,45_	*p*	η_p_^2^	*F*_1,45_	*p*	η_p_^2^	*F*_1,45_	*p*	η_p_^2^
**A**_**c**_	6.4	.02*	.12	.6	.43	.01	.7	.39	.01
**π**_**c**_	.6	.45	.01	.7	.41	.01	.5	.47	.01
***a***	.2	.66	< .01	.2	.65	< .01	< .1	.99	< .01
***b***	.7	.40	.02	.3	.59	< .01	< .1	.91	< .01

* : *p* < .05

### Exploratory movements

**Fig 4:**
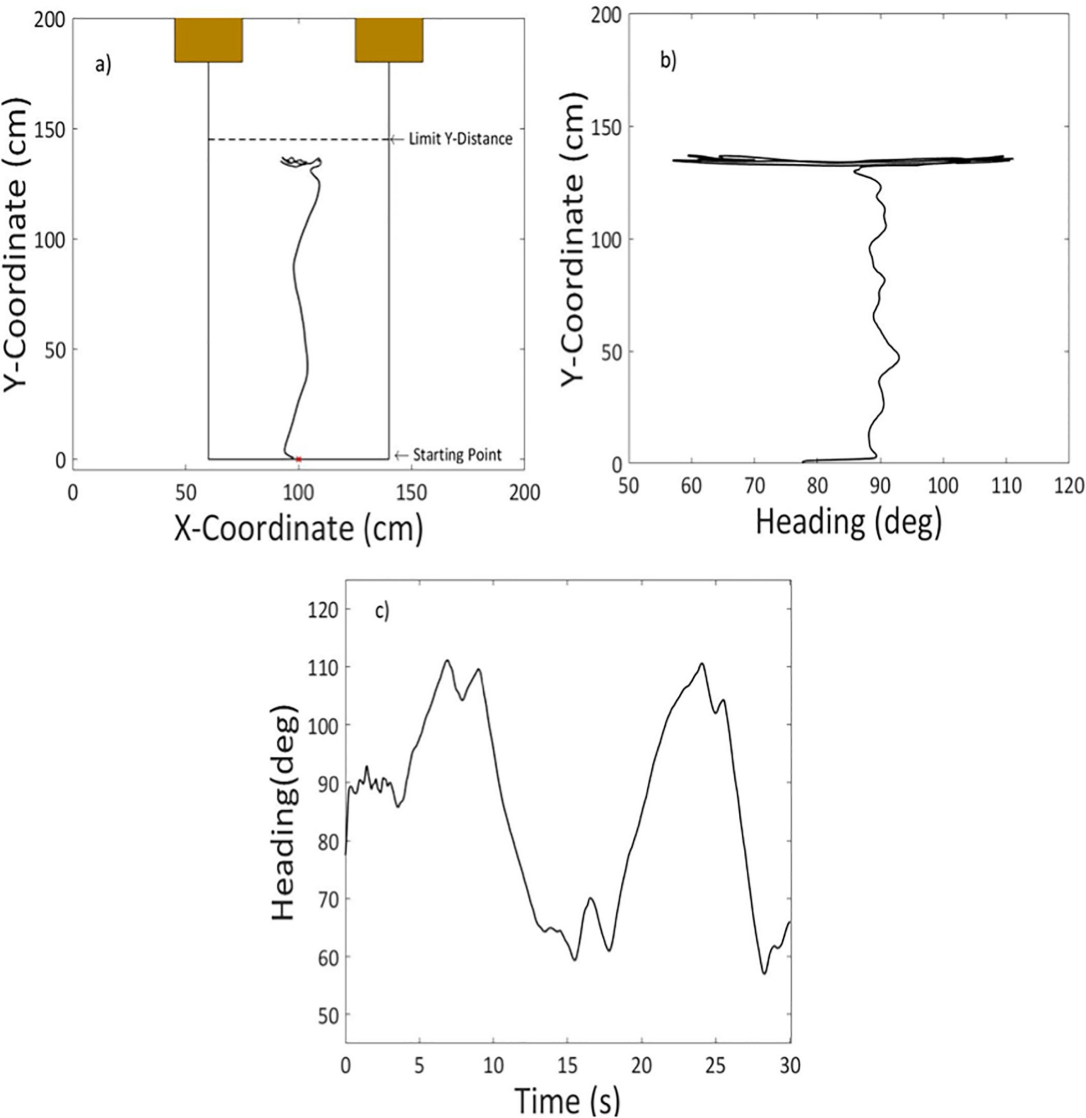
Exploratory movements in a representative trial. A. Starting point, allowed limit in *y* position, and participant’s translation. B. Time-series of *y* position plotted as a function of heading angle. C. Heading angle as a function of time.

Fig 4 presents a one-trial example of the exploratory movements. Fig 4A shows the *y* coordinate versus the *x* coordinate, Fig 4B the *y* coordinate versus the heading direction, and Fig 4C the heading direction over time. Overall, the figure illustrates the typical behavior in which the participant first approached the aperture and then showed relatively large exploratory oscillations in the heading direction (see also S1 Video). Remember from the Methods section that our analyses focused on the final exploratory phase.

Table 2 presents the results of the ANOVAs on the linear displacement, angular displacement, minimal distance to the aperture, and average number of active motors. The independent variables in these analyses were aperture width, shoulder width, and practice condition. All four dependent variables showed a highly significant dependence on aperture width. The only other significant effect in Table 2 was the one of training condition on linear displacement.

**Table 2:** Results of ANOVAs on the exploratory movement variables.

	Linear Displacement			Angular Displacement			Minimal Distance			Active Motors		
	*F*	*p*	η_p_^2^	*F*	*p*	η_p_^2^	*F*	*p*	η_p_^2^	*F*	*p*	η_p_^2^
**A**	8.2	***	.16	13.9	***	.24	7.4	***	.14	48.4	***	.79
**S**	1.6	.21	.04	.2	.69	< .01	< .1	.76	< .01	< .1	.92	< .01
**T**	9.7	**	.18	.9	.35	.02	2.4	.13	.05	< .1	.93	< .01
**A x S**	.5	.76	.01	.4	.86	< .01	.6	.74	.01	.7	.59	.02
**A x T**	1.6	.18	.03	.9	.50	.02	1.6	.14	.03	.8	.55	.02
**S x T**	1.0	.34	.02	.3	.60	< .01	1.5	.23	.03	.8	.38	.02
**A x S x T**	.2	.96	< .01	1.1	.35	.02	.8	.59	.02	.5	.77	.01

A: Aperture width; S: Shoulder width; T: Training.

**: *p* <. 01

***: *p* < .001. For effects and interactions that imply a within-subjects factor (A, A x S, A x T, and A x S x T), the degrees of freedom of the effect and the error were 6 and 270, respectively; for the remaining effects and interactions (S, T, and S x T), these degrees of freedom were 1 and 45.

**Fig 5:**
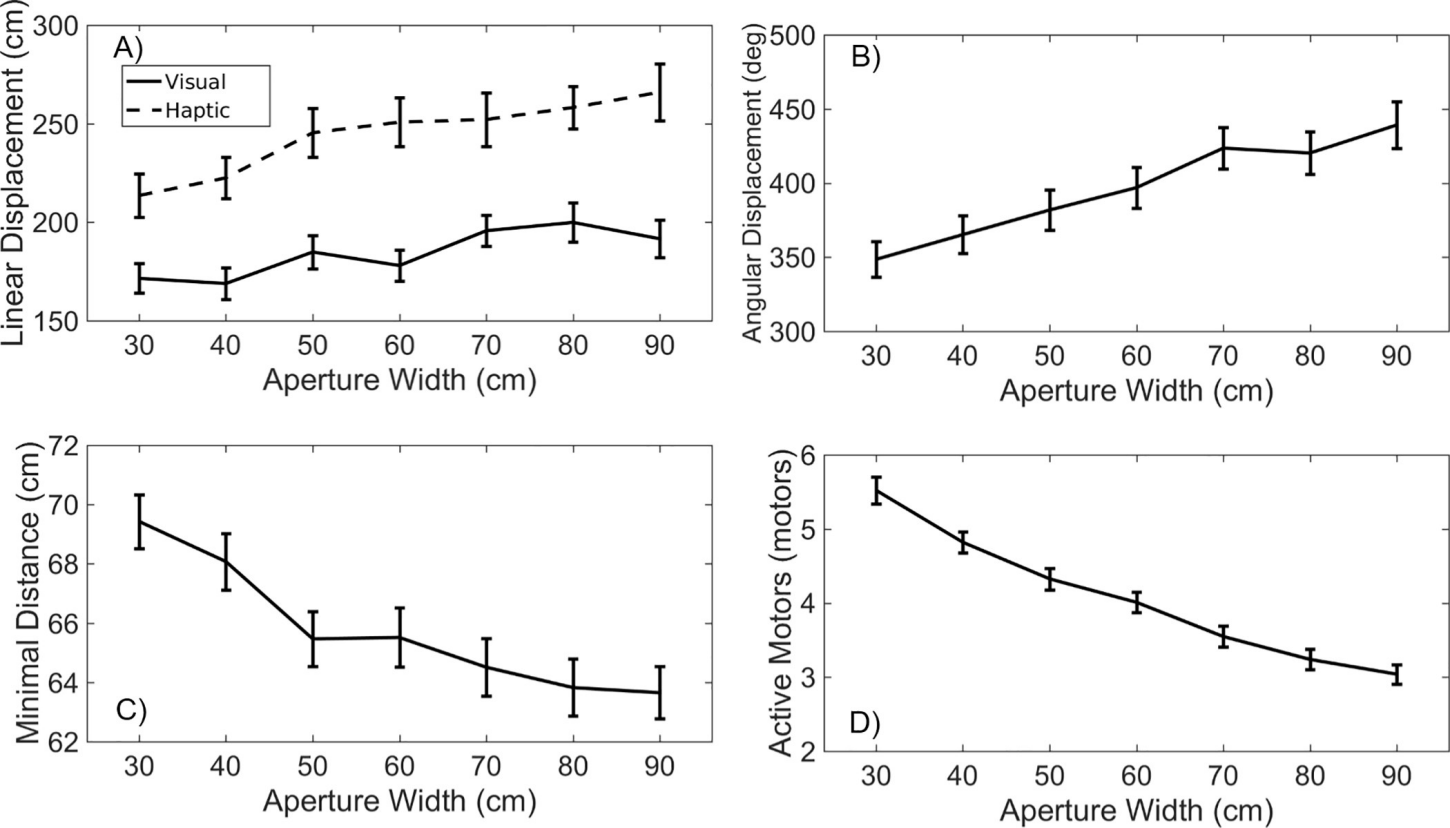
Movement variables as a function of aperture width. A. Linear displacement (for visual and haptic training groups). B. Angular displacement. C. Minimum distance. D. Average number of active motors. Error bars represent standard errors of the mean (SEM).

Fig 5 shows the direction of the significant effects. Fig 5A and 5B indicate that the linear and angular displacements increased with increasing aperture width. On the contrary, Fig 5C and 5D show that the minimal distance and the average number of active motors decreased with increasing aperture width. Taken together, these effects indicate a strong relation between the exploratory movements and the aperture width: any of the variables that we considered to characterize the exploratory movements (or the vibratory stimulation obtained through them) provided a clear indication about the width of the aperture in front of which the exploratory movements were performed. In addition, Fig 5A shows that participants who used a rod in the practice trials covered a larger linear distance than participants who performed the practice trials with vision.

### Type of errors

Table 3 presents the results of the ANOVAs with the type of error (false positive errors, correct judgments, and false negative errors) as independent variable. The dependent variables in these analyses were the aperture-normalized versions of the linear and angular displacements, the minimal distance to the aperture, and the average number of activated motors. As can be seen in the table, all these variables were significantly related to the type of error.

**Table 3:** Results of ANOVA on aperture-normalized exploratory movement variables.

	Linear Displacement			Angular Displacement			Minimal Distance			Active Motors		
	*F*_2,60_	*p*	η_p_^2^	*F*_2,60_	*p*	η_p_^2^	*F*_2,60_	*p*	η_p_^2^	*F*_2,60_	*p*	η_p_^2^
**Error Type**	18.1	***	.38	12.8	***	.30	63.6	***	.68	12.2	***	.29

***: *p* < .001.

**Fig 6:**
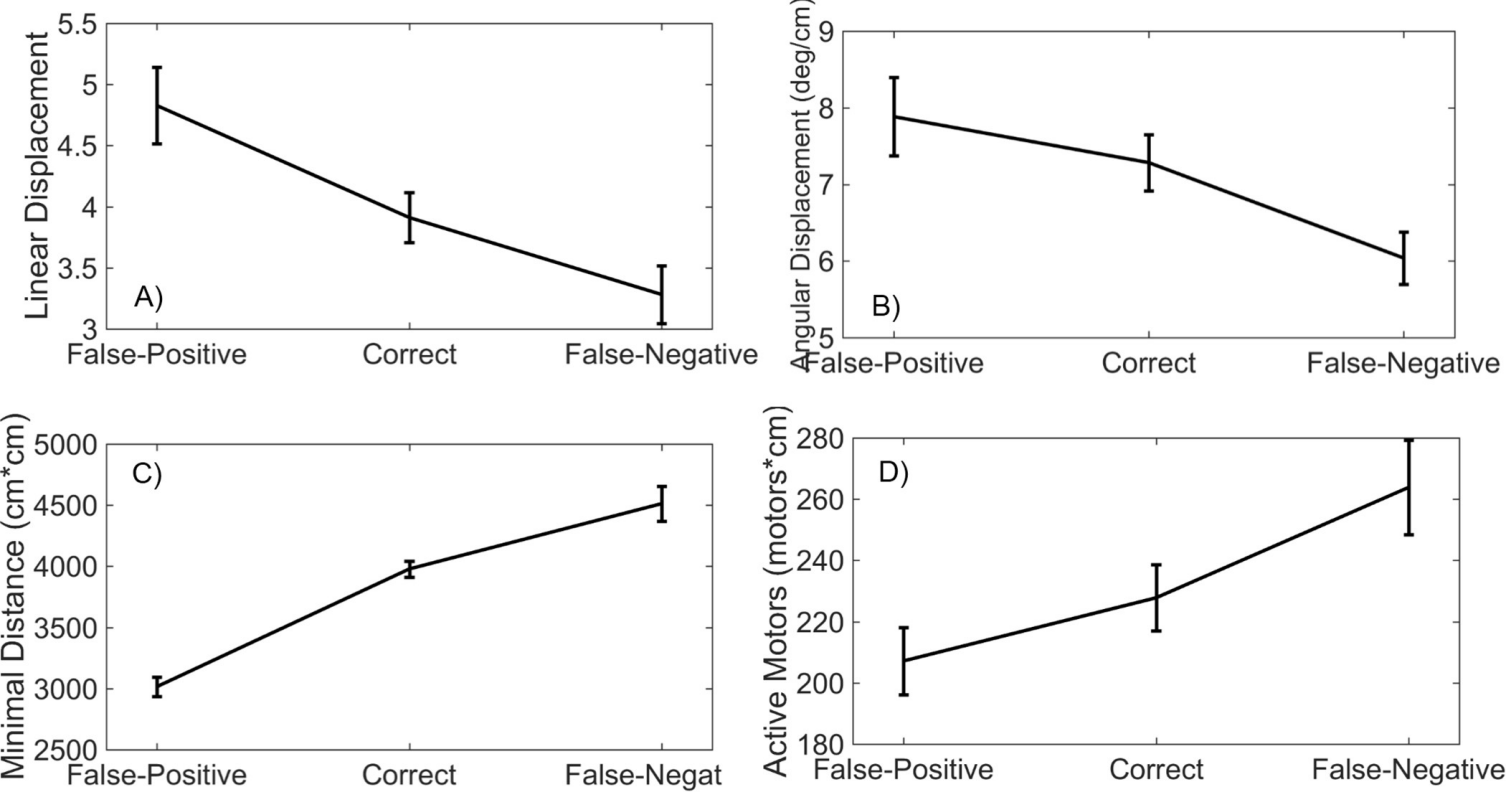
Aperture-normalized exploratory variables as a function of type of error. A. Linear displacement. B. Angular displacement. C. Minimum distance. D. Average number of active motors. Error bars represent standard errors of the mean (SEM).

Fig 6 shows the averages of the dependent variables per error condition. False-positive judgments went together with more linear and angular displacement than false-negative judgments (Fig 6A and 6B). In contrast, false-positive judgments went together with a smaller minimal distance to the aperture than false-negative judgments (Fig 6C). This may be related to Fig 6A and 6B in the sense that if one further approaches the aperture, more linear and angular exploration is needed to detect the edges of the aperture. Finally, false-positive judgments also went together with a smaller average number of active motors than false-negative judgments (Fig 6D).

## Discussion

The present study applied the theory of affordances in the field of sensory substitution. Our first purpose was to demonstrate the body-scaled nature of SSD-based perception, using the perception of passability as task. We analyzed two groups of individuals, with narrow and wide shoulders. Those with narrow shoulders judged narrower apertures as passable than those with wide shoulders. This difference disappeared when aperture width was scaled to shoulder width. The critical value of the dimensionless number, π_c_, defined as the value that leads to 50% of affirmative judgments, did not differ for the groups with narrow and wide shoulders. Furthermore, the overall π_c_ value of 1.22 that we obtained for SSD-based perception was not significantly different from the value of 1.16 that Warren and Whang [35] reported for the visual perception of passability. The standard interpretation of such findings is that perception is scaled to the anthropometric dimensions of perceivers (e.g., [26, 29, 31–34, 43]).

A second purpose of the study was to analyze the exploratory movements that underlie the SSD-based perception of apertures. The amount of exploration increased as a function of the width of the apertures (Fig 5A and 5B). This pattern of results is consistent with a strategy in which individuals move the field of sensitivity of the SSD from one side of the aperture to the other side until they receive the stimulation from the edges of the aperture. Such a strategy is interesting from a theoretical perspective because it makes the exploratory movements themselves informative about the apertures. Rather than on the pattern of vibrotactile stimulation, participants may have based their judgments on the exploratory movements themselves. Related to this, false positive errors were associated to more pronounced exploratory movements and false negative errors to less pronounced exploratory movements (Fig 6A and 6B). These effects were mediated by the distance from which the apertures were explored: when the apertures were explored from a larger distance, less linear and angular exploration was needed to detect the edges, leading participants to perceive the apertures as smaller than they actually were, and vice versa.

The actual aperture widths were also related to the vibrotactile stimulation that participants received as a consequence of the exploration. On average, less motors vibrated when wider apertures were explored (Fig 5D). As was the case with the exploratory movements, the amount of vibrotactile stimulation was related to the type of errors (Fig 6D). This means that, instead of on the exploratory movements, or in addition to them, participants may have based their judgments on the vibrotactile stimulation received through the SSD. As a consequence, our results do not allow us to disentangle the precise roles of the exploratory movements themselves and the vibrotactile sensory flow that was obtained from the interaction of those movements and the environment. In any case, from previous research it is clear that which informational variables are detected is related to the exploratory movements that are performed [46], hence emphasizing the importance of analyzing the exploratory movements.

A third purpose of the study was to compare the effect of two brief practice conditions in which individuals used the SSD either with vision or with a short rod. The only significant practice-related effect that was observed in the experimental trials was related to perceptual exploration (Fig 5A). Our results indicate that, if anything, practice without vision leads to more extensive exploration. The finding that familiarizing oneself with an SSD works as well without vision as with vision (or better, [18]) may be related to the guidance hypothesis [47, 48]. That is, if users of an SSD rely too much on vision during the familiarization, they may show a drop in performance when vision is no longer available. This drop in performance, in turn, may be related to the fact that users who practice with vision do not sufficiently attune their attention to the relevant non-visual variables that are made available through the SSD (cf. [49, 50]).

It is interesting to relate our findings to a theoretical debate within ecological psychology. This debate concerns the theory proposed by Stoffregen and Bardy ([51]; cf. [52, 53]). The classic ecological position is that affordances can be specified by higher-order ambient energy patterns, which may be confined to a single type of ambient energy (such as light, sound, or the gravito-inertial energy array) or may span multiple types of energy [22]. Stoffregen and Bardy [51], in contrast, claim that specificity exists exclusively in the global array (i.e., in higher-order patterns that span multiple types of ambient energy). This debate is particularly relevant to the field of sensory substitution. Visually impaired individuals have access to a limited part of the global array, and one may want to ask to what extent this affects their ability to detect information that specifies their affordances [54].

In our experiment, participants perceived the body-scaled aperture width, making one judgment per trial. The question therefore is whether and how information about aperture width was detected during the 30 s of exploration that occurred in each trial. As mentioned earlier in this Discussion, Fig 5 shows that the exploratory movements themselves (and hence information in the kinaesthetic array) as well as the vibrotactile stimulation were closely related to the actual aperture widths. However plausible the use of global information patterns may be, in our interpretation such results are consistent with the traditional ecological claim that specifying informational variables may also be found in individual energy arrays (cf. [55–56]). Also note that the capability of individuals to detect specifying information has been questioned particularly for novel tasks situations, as in the present experiment [50, 57].

A recent study by Riehm et al. [57] may be helpful with regard to further questions concerning information usage (cf. [43]). Riehm et al. demonstrated that the auditory perception of passability is body scaled. In addition, they described a candidate source of information for aperture width, based on the direction of the edges of the aperture at different distances (their Figs 2 and 3). Given that many SSDs allow users to detect the direction of those edges, the source of information described by Riehm et al. may also be relevant for SSD-based research. To test the use of the suggested information, Riehm et al. manipulated the gain of the auditory information with regard to head rotations. In a possible experiment performed with an SSD that is similar to ours, such a manipulation would correspond to manipulating the gain between body rotations and the corresponding vibrotactile sensory flow. It is interesting to speculate that future experiments with such manipulations may shed light on the entangled contributions of exploratory movements and sensory flows in information detection.

As described above, the present research was inspired by the theory of affordances, which is a key part of the ecological approach to perception and action [22–25]. We do not claim that our results contradict alternative theories. Examples of such alternative theories may claim that perception entails the enrichment of ambiguous ambient energy patterns by means of, say, unconscious inference, statistical weighting, or use of internal representations and memory [58]. In fact, we believe that evidence that directly proves or disproves theoretical frameworks does not exist (cf. [59]), and hence that theories should be evaluated with regard to their elegance and parsimony or with regard to the research questions and hypotheses that they generate. In this sense one should note that many applications of the theory of affordances in sensory substitutions are possible. To mention one, in our laboratory we have recently designed and tested an SSD that is attached to the hand. This SSD detects distances to nearby objects in the pointing direction of the fingers and transforms them into vibrations. Preliminary experiments have shown that users of this SSD are rather successful in reaching and grasping tasks. In analogy to the present study, one may ask whether the judgments of graspability made with this SSD are body scaled in the same way as visual judgments of graspability (e.g., [60]).

More generally, our belief is that experiments and SSDs inspired by the theory of affordances may advance research in the field of sensory substitution for at least three reasons. First, given that affordances refer to possibilities for action, experiments about affordances by definition concern issues that are relevant to the control of action. Second, as properties of the environment, affordances are not associated to particular sense modalities. In fact, ecological psychologists often argue that affordances can be perceived by means of different perceptual systems. This is an elegant starting point for endeavors that aim to test if affordances can be perceived through novel perceptual systems created with SSDs. Finally, affordances are perceived through exploratory actions [22]. This means that an affordance-based approach naturally leads to an increased focus on exploratory behavior, which, as we have argued throughout this article, is a crucial aspect of sensory substitution.

## Supporting information

S1 Video Single-trial example of functioning of SSD and time-evolution of crucial variables. (MOV)

S1 Dataset (XLSX)
